# Experimental Exposure to Bisphenol A Has Minimal Effects on Bone Tissue in Growing Rams—A Preliminary Study

**DOI:** 10.3390/ani12172179

**Published:** 2022-08-25

**Authors:** Jana Brankovič, Jakob Leskovec, Sabina Šturm, Vesna Cerkvenik-Flajs, Saša Šterpin, Joško Osredkar, Estera Pogorevc, David Antolinc, Milka Vrecl

**Affiliations:** 1 Institute of Preclinical Sciences, Veterinary Faculty, University of Ljubljana, Gerbičeva 60, 1000 Ljubljana, Slovenia; 2 Department of Animal Science, Biotechnical Faculty, University of Ljubljana, Groblje 3, 1230 Domžale, Slovenia; 3 Institute of Pathology, Wild Animals, Fish and Bees, Veterinary Faculty, University of Ljubljana, Gerbičeva 60, 1000 Ljubljana, Slovenia; 4 University Institute of Clinical Chemistry and Biochemistry, University Medical Centre Ljubljana, Zaloška Cesta 2, 1000 Ljubljana, Slovenia; 5 Faculty of Pharmacy, University of Ljubljana, Aškerčeva 7, 1000 Ljubljana, Slovenia; 6 Small Animal Clinic, Veterinary Faculty, University of Ljubljana, Cesta v Mestni log 47, 1000 Ljubljana, Slovenia; 7 Chair for Testing in Materials and Structures, Faculty of Civil and Geodetic Engineering, University of Ljubljana, Jamova 2, 1000 Ljubljana, Slovenia

**Keywords:** bisphenol A, rams, serum bone biomarkers, bone geometry, trabecular bone, cortical bone, bone biomechanics, mineral content

## Abstract

**Simple Summary:**

Bisphenol A (BPA) is a well-known synthetic compound that belongs to the group of chemicals that disrupt the endocrine system in humans and animals. Although bones represent a potential target for these compounds, studies investigating BPA-related effects in bones in large farm animals are limited. We exposed young rams aged 9–12 months to BPA through feed for 64 days and investigated the effects of BPA on bone length, mass, microscopic structure, mineral content, strength, and serum bone parameters. We discovered that BPA had no significant effects on most of the parameters studied. Only manganese was decreased, and copper increased in the femurs of the BPA-exposed rams. These results suggest that a 2-month, low-dose exposure to BPA in growing rams did not affect the macro- and microstructure, metabolism, and biomechanical behavior of femur bones; however, it did affect the composition of microelements in bone, which could affect the bone in the long term.

**Abstract:**

Bisphenol A (BPA) is a well-known synthetic compound that belongs to the group of endocrine-disrupting chemicals. Although bone tissue is a target for these compounds, studies on BPA-related effects on bone morphology in farm animals are limited. In this preliminary study, we investigated the effects of short-term dietary BPA exposure on femoral morphology, metabolism, mineral content, and biomechanical behavior in rams aged 9–12 months. Fourteen rams of the Istrian Pramenka breed were randomly divided into a BPA group and a control group (seven rams/group) and exposed to 25 µg BPA/kg bw for 64 days in feed. Blood was collected for determination of bone turnover markers (procollagen N-terminal propeptide, C-terminal telopeptide), and femurs were assessed via computed tomography, histomorphometry, three-point bending test, and mineral analysis. BPA had no significant effects on most of the parameters studied. Only mineral analysis showed decreased manganese (50%; *p* ≤ 0.05) and increased copper content (25%; *p* ≤ 0.05) in the femurs of BPA-exposed rams. These results suggest that a 2-month, low-dose exposure to BPA in growing rams did not affect the histomorphology, metabolism, and biomechanical behavior of femurs; however, it affected the composition of microelements, which could affect the histometric and biophysical properties of bone in the long term.

## 1. Introduction

Bisphenol A (BPA) is one of the most widely produced and used synthetic compounds in the world and belongs to the group of endocrine-disrupting chemicals (EDCs) [1]. BPA is used in the plastics industry, (thermal) paper, toys, electronic devices, water pipes, food contact materials, and dental materials; therefore, it is inevitable that humans are exposed to BPA daily [2]. The estimated daily exposure *per os* of the human organism to BPA is 0.48–1.6 µg/kg body weight (bw) [3]. In 2015, the European Food Safety Authority (EFSA) established a temporary tolerable daily intake (t-TDI) for BPA of 4 μg/kg bw/day, which was previously 50 μg/kg bw/day [4]. However, in the current re-evaluation and draft opinion, EFSA proposes to substantially reduce the TDI to 0.04 ng/kg bw/day [5]. This compound does not accumulate in the body; greater than 90% is excreted in the urine as metabolites within 24 h [6]. In the United States, BPA was detected in the urine of 92.6% of participants (4.5 µg/L in children aged 6–11 years, 2.5 µg/L in adults over 20 years) [7], whereas lower levels, i.e., 1.49 µg/L, were detected in the urine in Germany [8] and 2.55 µg/L in Belgium [9]. Conjugated BPA levels were determined in the blood of pregnant (4 µg/L) and nonpregnant women (1 µg/L) [10], human adipose tissue (3.78 ng/g), liver (1.48 ng/g), and brain (0.91 ng/g) [11].

The endocrine-disrupting activity of BPA has been described through several pathways with an affinity for binding to receptors, such as estrogen (ER) [12,13,14], androgen [15], aryl hydrocarbon receptor [16], or steroid and xenobiotic receptors [17]. This binding can disrupt endocrine system function in animals [3,18,19]. Although some studies assumed that greater than 1000-fold higher BPA concentrations produced the same effects as 17β-estradiol [12], others demonstrated that BPA is a potent xenoestrogen with biological effects at extremely low doses [19]. Reports of BPA interfering with the function of the endocrine system have been discussed in several articles [1,2,20]. Bone metabolism is sensitive to EDCs, including BPA, by exerting effects on the processes of bone formation and turnover [21]. Bone growth at the epiphyseal growth plate (EGP) during the intense pubertal growth spurt depends on genetic, nutritional, environmental, and hormonal factors, in which estrogens play an important role [22,23]. In the last decade, several studies have evaluated the effects of BPA on bone tissue in laboratory rodents. Offspring exposed to environmentally relevant BPA concentrations of 25 µg BPA/kg bw/day during gestation and early postnatal life via their mothers exhibited sex-specific changes in femur geometry but no changes in bone strength [24,25,26]. Recent studies by Lind et al. also showed sex-specific effects at doses well below the t-TDI of 4 μg/kg bw/day [27,28]. Developmental exposure via mothers of 0.5 µg/kg bw/day resulted in reduced bone geometry in male offspring at 5 weeks [28], which then normalized by 52 weeks of age [27]. However, changes in plasma levels of procollagen type I N-terminal propeptide (P1NP, also PINP), bone stiffness, bone marrow, and inflammatory plasma markers persisted in female offspring [27]. Dirkes et al. reported developmental BPA effects in adult male mouse offspring [29]. In farm animals, BPA was detected in fresh pork loin meat under standard stock-farming conditions (in a control group of pigs) [30]. A study in ruminants showed that BPA is a potential EDC in goats and confirmed the susceptibility of livestock to adverse effects of EDCs on the endocrine and reproductive systems [31]. Studies on the effects of BPA in large laboratory animals (translational studies) are scarce. In pigs, the effects of BPA on the reproductive system and intrahepatic nerve fibers have recently been studied [32,33]. To our knowledge, there are no studies in ruminants investigating the impairment of bone tissue by dietary BPA intake. In sheep, an environmental EDC, polychlorinated biphenyls (PCBs) 118 and 153, decreased bone geometry and mineral content in female sheep fetuses but not in male fetuses or adult sheep after 4-months in utero or oral exposure, respectively [34]. Sewage sludge from fertilized pastures containing environmental contaminants, including PCBs, also impaired bone tissue homeostasis in sheep, especially in males, after 18 months of in utero and later oral exposure [35]. Our own studies in rat offspring lactationally exposed to PCBs 155 and 169 revealed congener- and age-dependent effects on the femur growth rate, geometry, biomechanics, mineral composition, and histomorphometric characteristics [36,37,38]. In adult and fetal human bone tissue, BPA disrupted bone homeostasis in osteoblasts but not osteoclasts [17]. Kim et al. demonstrated that BPA and its alternatives, bisphenol F and tetramethyl bisphenol F (TMBPF), are not toxic to osteoclast differentiation in vitro; in fact, TMBPF enhanced this process [39]. Recent studies on BPA also reported its effects on osteocytes (inhibited cell viability, apoptosis, and pyroptotic death) that potentially adversely contribute to the development and metabolism of bone [40,41].

Therefore, the aim of this preliminary oral toxicity study, which lasted 64 days using an acute, repeated daily dose of BPA, was to investigate the possible BPA-related effects on bone tissue and its metabolism in young, growing male sheep. It was the first study on the effects of BPA on bone tissue in sheep. The selection of a particular animal model was based on its presumed suitability for translational studies. Sheep are suggested as a suitable model for experimental orthopedic studies in humans, given their similarities in bone biochemistry and mineral composition, trabecular bone structure, modeling, and biomechanics. Most ossification centers of long bones close between 12 and 30 months of age (the distal center of the femur between 18 and 26 months of age) [42,43,44]. Thus, the study was conducted in rams aged 9–12 months with active EGP. The following parameters were assessed: bone turnover using serum bone markers and geometry, density of cortical and trabecular bone tissue, EGP architecture, mineral content, and biomechanical behavior.

## 2. Materials and Methods

### 2.1. Study Design

#### 2.1.1. Animal Housing

To evaluate the effects of BPA on bone parameters, an in vivo animal study was performed on 14 rams *(Ovis aries)* of the autochthonous Slovenian sheep breed Istrian Pramenka in the western part of Slovenia with a moderate continental climate. At the Infrastructure Centre for Sustainable Recultivation (ICSR) in Vremščica, Slovenia, the rams were born (average body mass at birth of 4.36 ± 0.7 kg), individually marked with conventional sheep ear tags, selected for the study, and housed in a roofed stable with concrete paving for 9 months until the start of the procedure, which lasted from October to December. The sample size for the procedure was determined according to guideline VICH GL48 [45]. Rams were housed in a deep bedding system (straw, hay) and fed *ad libitum* with hay and once daily with 550 g of commercial pellets (Schafkorn, Raiffeisengenossenschaft Osttirol, Lienz, Austria, a certificate of ingredients and chemical composition is provided in the Appendix) from stainless steel bowls. They had unlimited access to drinking water from concrete rainwater tanks provided in enamel bowls (50 L). Feeding management was the same for all rams. A keeper monitored the animals’ condition thrice daily. The animals entering the procedure were clinically healthy. Basic blood analysis was performed before the procedure; blood was collected in the morning before pellets were administered [46]. Vital signs (body temperature, pulse, respiratory rate, ruminations) and serum biochemistry parameters (Supplementary Table S1) were consistent with a previous study of this breed [47].

#### 2.1.2. Chemicals

Chemicals and their preparation were previously reported by Cerkvenik-Flajs et al. [48]. Briefly, once daily, BPA (certified reference standard of ≥99.0% analytical purity, Sigma-Aldrich, Merck, Darmstadt, Germany, dissolved in anhydrous ethanol at 2.5 mg/mL) was administered to the treated group in feed at a dose of 25 µg/kg bw, while a control group received 1 mL of ethanol daily in feed with no BPA [48]. During the study, the solutions were stored at ambient temperature in sealed amber glass bottles. The BPA dose used (25 µg/kg bw/day) was based on the assumptions of Guignard et al., who suggested that people could ingest several tens of µg/kg BPA per day [49] based on previous pharmacokinetic studies performed on various animals [50] and the commonly described human plasma BPA concentrations in the range of ng/mL [3,4,10].

#### 2.1.3. Procedure Design

The rams were randomly divided into two experimental groups (physical randomization using withdrawn papers), a BPA group and a control group (7 rams/group). One day before the first BPA administration at 9–10 months of age, the rams weighed 34.5–54 kg (mean body mass of 43.0 ± 4.9 kg). During the procedure, the animals of the two groups were housed in the stable mentioned previously, separated by 2 m with a wooden/stainless steel fence to prevent physical contact but still allow visual and auditory contact between the two groups. The rams had hay and straw bedding cleaned weekly and were exposed to a natural light/dark cycle at a temperature of 6 to 15 °C and relative humidity of 45–55% (Renkforce, Conrad Electronic SE, Hirschau, Germany). Rams were weighed once per week during the BPA treatment. Each morning, all rams were sorted into individual wooden boxes for pellet feeding. The daily amount of BPA for individual rams was prepared according to body mass. After complete BPA impregnation of the pellets, the pellets were offered to the rams, and it was ensured that all rams had eaten their entire ration. After the meal, rams were released back to group housing, where the water regime was the same as mentioned before. On Day 31 of the study, blood samples were collected in the morning before pellet administration. The treatment lasted 64 days. Before anesthesia and euthanasia (age 11–12 months), the rams were weighed, and blood was collected. The rams were premedicated with xylazine i/v (2 mL Xylased 5%, Chanelle Pharmaceuticals Ltd., Loughrea, Ireland) and euthanized after 7–10 min with pentobarbital (Exagon, Richter Pharma, Wels, Austria; 2 mL/10 kg bw). After euthanasia, we dissected the left and right femur bones for further analysis and collected the liver and kidneys for the study of edible tissues [48,51]. The same veterinarian checked the health status of the animals throughout the procedure, weighed the animals weekly, obtained blood samples, and performed anesthesia and euthanasia at the end of the procedure.

Throughout the procedure, extreme care was taken to ensure that the animals were not exposed to uncontrolled BPA exposure from products, i.e., glass or BPA-free materials were used [52]. The absence of BPA contamination in water, pellets, and hay was verified [51].

#### 2.1.4. Approval of the Procedure on Animals

The animal procedure was approved by the Administration of the Republic of Slovenia for Food safety, Veterinary, and Plant protection (approval numbers U34401-3/2015/8 and U34401-3/2015/17). Animal husbandry and animal handling procedures complied with the Slovenian Animal Protection Act (Official Gazette of the Republic of Slovenia 38/2013) [53], Council Directive 2010/63/EU [54], and ethical principles. Humane care of the animals was ensured throughout the study [55].

### 2.2. Analyses of BPA in Blood, Liver, and Kidney

Previously published studies on these rams demonstrated the average maximum plasma concentration of total BPA in the blood plasma of rams from the BPA group was 10.93 µg/L, measured after the first administration [51]. In the same group, significant BPA concentrations were found in the liver and kidney, where BPA was metabolized and eliminated, with mean ± SD concentrations of the aglycone form of 1.01 ± 0.71 µg/kg and 0.59 ± 0.45 µg/kg, respectively, and of total BPA of 4.70 ± 1.34 µg/kg and 16.62 ± 4.78 µg/kg, respectively [50]. The concentrations in the liver and kidney were measured 3–6 h after the last dose was administered on Day 64.

### 2.3. Analyses of Bone Turnover Markers in Blood Serum

Blood was collected from the jugular vein in the morning (9–10 AM), before pellet feeding [46], and three times during the procedure (a day before the first BPA exposure, on Day 31, and at the end of the procedure on Day 64 of BPA exposure). It was collected in glass heparinized vacuum tubes [51] containing no anticoagulant, centrifuged within 30 min of collection (3000× *g* for 10 min), and stored at −20 °C during transport and at −80 °C until analysis. Serum biochemistry to assess renal and hepatic function, i.e., levels of urea, creatinine, cholesterol, triglycerides, aspartate aminotransferase (AST), and gamma-glutamyl transferase (GGT) activity, as well as levels of calcium (Ca) and inorganic phosphate (PO_4_), were analyzed in addition to bone turnover markers, including C-terminal telopeptide of type I collagen (CTX-I) and P1NP. Serum basic biochemistry was determined using an automated chemical analyzer (Olympus Corp., Hamburg, Germany). Bone turnover markers were selected as previously reported [42,56], and commercially available kits were used to measure their serum levels, i.e., the Sheep Crosslaps (CR) Elisa Kit (MBS7240838, MyBioSource, CA, USA) and the Sheep Total Procollagen Type I Intact N-terminal Propeptide (TP1NP) Elisa Kit (MBS9358461, MyBioSource, San Diego, CA, USA). When the bone turnover marker CTX-I was analyzed, two serum samples (one sample per experimental group) were hemolytic and had to be excluded from further analysis. The ratio between Ca and P was calculated.

### 2.4. Bone Tissue Preparation

The right hind legs were disarticulated at the hip. The femur was dissected, and soft tissues were removed with a knife and a scalpel. The distal extremity of the right femur was cut with a saw at the metaphysis in a transverse plane approximately 1 cm proximal to the distal EGP, and the limb extremity was longitudinally halved and immediately fixed in 5% buffered formalin. The left hind leg was shaved and removed through the hip joint, and the thigh was dissected (femur along with the thigh musculature) and frozen at −20 °C until computed tomography (CT) scanning.

### 2.5. Femur Geometry and Microarchitecture of Bone Tissue

**Figure 1:**
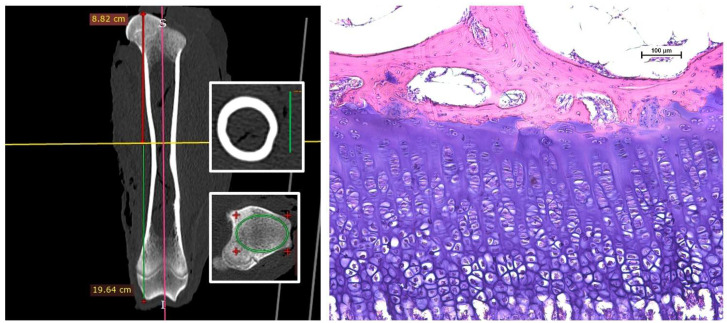
Representative images of macro- and microstructure of a ram’s femur at 12 months. (**left**) A CT image showing femur geometry. The longitudinal image shows femur length measurements; the insets show cross-sectional images of the diaphysis (upper inset) and epiphysis (lower inset) used for cortical and trabecular parameter analysis. (**right**) A photomicrograph used for histomorphometric analysis of the structure and zones of the distal epiphyseal growth plate (EGP), hematoxylin and eosin staining, scale bar = 100 µm. RZ—zone of reserve cartilage, PZ—zone of proliferation, HZ—zone of hypertrophy, CZ—zone of calcified cartilage.

The geometry of the femur was determined by scanning frozen left thighs using a Siemens Somatom Scope CT scanner (Siemens, Erlangen, Germany). Acquisition and reconstruction protocols were established before scanning and were the same for all specimens: the voltage used was 130 kV, automatic tube current modulation was 31 mAs, detector collimation was 16 × 0.6 mm, slice thickness was 1 mm with a 0.8-mm recon increment, and reconstruction core kernel B70s sharp was used. The threshold of 1700 to 2000 cm^−1^ was used to define cortical bone, and 300–600 cm^−1^ was used to define trabecular bone. Femur length was measured in the sagittal plane from the femoral head to the medial condyle, and femur width was measured in the axial plane at 50% of the femur length (RadiAnt DICOM Viewer^©^ 4.6.9, Medixant, Poznan, Poland) as previously described [38]. The medullary cross-sectional area and total cross-sectional area (tCSA) were measured using ImageJ/Fiji 1.43 software [57] at two different locations: at 50% of the femur length for geometry purposes and at the point where the bones fractured during the three-point bending test for biomechanical analyses. Cortical CSA (ctCSA) and the ctCSA/tCSA ratio were calculated. Cortical and trabecular bone density were measured using a 1-mm slice in the axial plane (RadiAnt DICOM Viewer^©^ 4.6.9). Trabecular bone density was determined 1 cm proximal to the distal EGP, and cortical bone density was determined at 50% of the femoral length. Other trabecular microarchitecture parameters were analyzed proximal to the distal EGP using the BoneJ plug-in for ImageJ software [58]: bone volume fraction, i.e., ratio of bone volume (BV) to total volume (TV), mean trabecular thickness (Tb.Th, mm), mean trabecular separation (Tb.Sp, mm), and degree of anisotropy (DA). Representative CT images for analyzing bone geometry and microarchitecture are presented in Figure 1 (left panel).

### 2.6. Femur Histomorphometry

After fixation in formalin, the longitudinally bisected distal extremities of the right femurs were decalcified in Osteosoft^®^ solution (Merck, Darmstadt, Germany) for 6–7 weeks at room temperature until the bone tissue was sufficiently demineralized and then embedded in paraffin blocks (Tissue-Tek^®^, Sakura Finetek, Torrance, CA, USA, Europe). Tissue sections of 5 µm (microtome Leica SM2000R, Nussloch, Germany) that were cut longitudinally through the EGP and metaphysis were stained with hematoxylin and eosin stain (HE) and cover slipped (Gemini AS slide stainer and cover slipper ClearVue, Thermo Fisher Scientific, Cambridge, UK). Comparable longitudinal sections were used for histomorphometric analysis (*n* = 1 section/sample). Images were obtained by a light microscope (Eclipse Ni-U, Nikon, Tokyo, Japan) and a digital camera (DS-Fi1, Nikon, Tokyo, Japan) and quantified with the imaging software program NIS-Elements BR 4.6 (Nikon Instruments Europe B.V., Amstelveen, The Netherlands). In analyzing the zones of the distal EGP, the relative thickness of the zones of the reserve, proliferative, hypertrophied, and calcified cartilage (RZ, PZ, HZ and CZ, respectively) were morphologically analyzed, i.e., measured in µm (*n* = 7 measurements/zone per sample), and calculated to the total thickness of EGP (in µm, excluding the zone of resorption), as previously described [36]. A representative micrograph for histomorphometric measurements is presented in Figure 1 (right panel).

### 2.7. Biomechanical Properties of the Femur

After CT, the left femurs were cleaned of soft tissues and weighed (Scale Kern PCB 2500-2, Balingen, Germany). Biomechanical properties were determined by a three-point bending test using a universal hydraulic testing machine MTS (Exceed, Model 43, MTS Systems Corporation, Eden Prairie, MN, USA). Each femur was loaded with a press head (diameter 20 mm) perpendicular to its longitudinal axis at the mid-diaphysis at a loading rate of 0.2 mm/s. The femurs were placed on two supports (diameter 5 mm) with a span of 120 mm between them. The caudal surface of the femur was facing upward when it was on the supports. During the conduction of the bending test, the applied displacement and corresponding force were recorded. Based on the obtained data, work to fracture, stiffness, modulus of elasticity, and bending strength (ultimate stress) were calculated as previously described [38]. The geometric properties of the bone cross-section at the mid-diaphysis were determined from CT scans.

### 2.8. Femur Mineral Content

To analyze the mineral content of the bone ash, the proximal extremity of the left femur was cut with a belt saw for bones at approximately 15% of the total length for further analysis. The analyses of dry matter and ash were performed by standard methods [59] with minor adaptations; the observed piece of bone was dried at 80 °C for 20 h and then at 104 °C for 3 h (SP50C dryer, Kambič, Semič, Slovenia). After drying, the bone was ashed at 550 °C for 8 h (L9/11, Nabertherm, Lilienthal, Germany) and then ground in a laboratory mill. The mineral composition of the ash was determined by standard methods of spectrophotometric determination (Cary 50 Probe, Varian, Palo Alto, CA, USA) for phosphorus (P) and atomic absorption spectroscopy (Aanalyst 200, Pelkin Elmer, Waltham, MA, USA) for Ca, magnesium (Mg), copper (Cu), zinc (Zn), iron (Fe), and manganese (Mn) as described previously [59]. The ratio between Ca and P was calculated.

### 2.9. Statistical Analysis

Data were analyzed using SAS software (ver. 9.4; SAS Institute Inc, Cary, NC, USA) with the Proc Mixed function. In the statistical model, the observed group was considered a fixed effect, except for the serum biochemical parameters, where the group, the time of observation, and their interaction were used as fixed effects, whereas repeated measurements within the animal were considered. The least square means are presented, and differences were determined by a Tukey–Kramer multiple comparison test. The dispersion is expressed as the standard deviation (SD) or the standard error of the mean (SEM), except in the two serum bone markers, where relative values were used. Statistical significance was considered when *p* ≤ 0.05.

## 3. Results

### 3.1. Serum Biochemistry and Markers of Bone Turnover

In the BPA group, urea and creatinine as renal function parameters and cholesterol, AST, and GGT activity as hepatic parameters showed no change compared with the control group within each blood collection during the procedure (a day before the first BPA treatment, on Day 31, or at the end of the procedure on Day 64 of BPA exposure), as shown in Table 1. Within the same experimental group, urea was decreased in the control group from Day 31 to 64 (*p* = 0.037), and AST was decreased in the BPA group on Day 31 (*p* = 0.022) but not Day 64. Creatinine was decreased and GGT was increased in the overall sample (disregarding the experimental group) on Day 31 (*p* = 0.026 and *p* = 0.297, respectively) and Day 64 (*p* = 0.022 and *p* = 0.006, respectively) compared to Day 0.

Analysis of Ca revealed no differences between the two experimental groups throughout the procedure, whereas the PO_4_ value decreased over time until Day 64 (though significantly only in the control group, *p* = 0.012). Thus, the ratio between Ca and P increased from Day 0 to Day 64 in the control group (*p* = 0.003). Serum concentrations of the markers CTX-I and P1NP in the rams were more heterogeneously distributed than suggested by the manufacturer (MyBioSource, San Diego, CA, USA, Table 2) and had to be transformed to relative values. Therefore, the results measured before the procedure served as an individual baseline value for each animal. The following values measured during the procedure were calculated relative to their baseline values. The CTX-I values showed a continuous increase until Day 64 (significant in the control group compared to the before treatment only, *p* = 0.047).

### 3.2. Femur and Liver Mass, Femur Geometry, and Histomorphometry

Relative liver mass (liver mass compared to bw) was not changed in the BPA group (1.58 ± 0.07, *n* = 7) compared with controls (1.58 ± 0.08, *n* = 7, *p* = 0.927). The relative femur mass (femur mass compared to bw), femur geometry, and histomorphometric parameters are summarized in Table 3. No difference in femur mass was observed between the experimental groups. Scans showed no change in femoral length, width, or cross-sectional areas between the two groups. At mid-diaphysis, cortical bone accounted for approximately 50% of the bone’s tCSA in both groups. The densities of both bone tissue types and the microarchitecture of the trabecular bone (volume fraction, trabecular parameters) were also comparable between the experimental groups. Due to technical problems in the decalcification process, one of the specimens in the control group was excluded from further processing and analysis. The thickness of EGP was not changed between the groups. In the BPA group, the relative thickness of the RZ, PZ, HZ, and CZ of the distal EGP did not change compared with the control group and was measured as 57.3 ± 12.7, 170.9 ± 19.9, 146.1 ± 29.7, and 31.2 ± 5.8 µm, respectively.

### 3.3. Femur Biomechanical Behavior and Mineral Content

The biomechanical and mineral properties of the left femur are summarized in Table 4. Comparison of the basic biomechanical parameters of the left femur (max force, displacement, work to fracture, stiffness, modulus of elasticity, and bending strength) obtained with the three-point bending test for the control and BPA groups showed no significant differences between the two groups. Additionally, between the observed groups, no major differences were observed in the content of ash, major minerals, and their ratio (i.e., Ca, P, Ca:P, and Mg), and two trace elements, Zn and Fe. The animals in the BPA group had a higher content of Cu (25%; *p* = 0.011) and substantially reduced Mn content (50%; *p* = 0.006) in the ash compared to the control group.

## 4. Discussion

This preliminary 2-month study investigated the effects of dietary exposure to an environmentally relevant dose of BPA on long bone metabolism. We analyzed bone turnover markers, femoral growth and geometry (the distal EGP and trabecular microarchitecture), and mineral content and investigated to what extent these parameters influence bone biomechanical behavior in young, growing rams, given the similarities of sheep bone anatomy, trabecular structure, and mineral metabolism to humans [44]. A two-month exposure to a daily dose of 25 µg BPA/kg bw/day showed no systemic or acute effects on rams, and this finding was also supported by relative liver mass and serum parameters of liver and kidney function, as they were in previously reported ranges for sheep breed Istrian Pramenka [47]. Serum and bone parameters of bone metabolism, mass, geometry (length, width, cross-sectional architecture), mineral content, and biomechanics seemed to be only slightly affected in the BPA group, suggesting no major biological effects in long bone tissues. Similar results were previously observed for ram body mass and reproductive parameters [51]. Rams’ internal exposure to BPA was detected in the BPA-exposed group, as previously reported in the Materials and Methods [48,51]. Concentrations in the liver and kidney were measured only a few hours after administering the last dose of the procedure, meaning that the concentrations in the tissues were probably temporary and not the result of any accumulation.

The serum creatinine and urea concentrations were slightly decreased but still in the physiological range at the end of the procedure (urea in the control group only), although the feed (protein) intake and final body mass of these ruminants were not altered throughout the procedure. The high serum PO_4_ concentration in all animals before BPA administration reflected their age and intense growth and possibly the daily administration of concentrated pellets. Therefore, the significant decrease in phosphate and increase in Ca:P ratio throughout the procedure—with increasing age—toward the Ca:P ratio common to adult animals, i.e., 1.37–1.86 [47], was expected and should not be considered an effect of BPA treatment. Decreased levels of AST and increased levels of GGT activity, although still in the physiological range (also reported when low doses of BPA were administered to rats [60]), could indicate a disturbance in biliary excretion [61], although the relative liver mass was not affected in the present study. Bone turnover markers showed a wide interanimal variation in this study (including preexperimental values). P1NP levels were expected to increase, reflecting increased bone formation with growth, whereas CTX-I levels indicate a bone resorption process [42]. After BPA exposure, stimulated proliferation and collagen accumulation could be expected in osteoblasts but not in osteoclasts [17], whereas negative effects such as apoptosis, pyroptotic death, and autophagy were induced in osteocytes [40,41]. Even when we tried to eliminate or control most of the possible disrupting factors (diet, age, sex, breed, housing, season, circadian rhythm, climate, time of blood collection, location and storage, methodology, prior validation) [46], their variability was significant. Similar observations have been reported in previous studies in sheep [35,42,43,62,63]. No effect on bone turnover markers was observed in lambs exposed to zeranol and ewes exposed to sewage sludge on pasture, despite differences in bone geometry [62], mineral density, and biomechanics [35]. Studies in bone turnover markers by Camassa et al. and Sousa et al. reported their large interindividual variability in serum levels [42,43,63]. In a 6-week BPA study in rats exposed to 0.5 µg/kg bw/day via drinking water, a change in P1NP was detected only in female offspring but not in males, whereas the CTX-I marker was not altered. On the contrary, bone geometry was altered only in males [28]. Therefore, the trend toward increased CTX-I without a concomitant change in P1NP *per se* was difficult to interpret. To reduce sample variability, urine samples of bone resorption markers should be considered together with the interpretation of blood markers [35].

As mentioned above, the daily administration of 25 µg BPA/kg bw for a period of 2 months did not appear to affect the growth of the femurs or the bone biomechanical behavior of the growing rams. The fact that no change was observed in bone size, bone mass, or both types of bone tissue at three different sites, i.e., the cortical bone at mid-diaphysis, the EGP structure at the metaphysis, and the trabecular bone adjacent to the metaphysis, further confirmed this assumption. The EGP zones of the distal metaphysis were appropriately thick and aligned, and the chondrocytes followed the pattern of secondary ossification. The mineral content (ash, Ca, P, and Mg) showed a similar composition for all tested bones. Given that the mineral composition of the bones is one of the main parameters for bone biomechanical behavior, the stiffness and bending strength obtained with the three-point bending test were, therefore, similar. Similar to our observations in males, sex differences were found in several recent studies [24,27,28,64]. In a study by Lejonklou et al., in rats exposed to 25 or 5000 μg BPA/kg bw/day, body mass and femur length did not change in male offspring as a function of the amount of BPA received, whereas femurs were elongated in female offspring. However, bone biomechanical properties were not altered [24]. Another recent BPA study on rats, corroborating our results, reported effects exclusively on the bones of female neonates exposed in utero (pregnant Wistar rats were administered 10 μg/kg/day intragastrically during gestation days 14–21) [64]. Additionally, at lower doses, sex-specific alterations were observed in adult animals. Male rat offspring exposed to a BPA dose of 0.5 µg/kg bw/day for 6 weeks exhibited reduced bone geometry at 5 weeks of age [28], which then normalized by 52 weeks of age [27]. However, in female offspring, changes, such as serum P1NP levels, reduced stiffness, and an increased number of fibrotic lesions in the bone marrow, were observed later in life [27,28]. Furthermore, the CTX-I marker was not altered in either sex [28]. In contrast to our outcomes, two recent studies reported adverse effects of BPA in adult male mice on trabecular and cortical bone tissue [29] and impaired bone stiffness and strength [29,65] compared to female adult offspring (exposed to 200 μg BPA/kg bw/day gestationally and lactationally). Considering the sexually dimorphic effects of BPA mentioned above, a comparison with ewes would be beneficial, as most reported BPA-related effects on bone tissue were found in females. Rodents were used in the studies mentioned above. In sheep, further sub-chronic studies would suggest a longer exposure time to repeated doses of BPA (90 days or longer) [66].

The amount of ash and the content of macrominerals did not differ; however, Mn and Cu showed significant differences between the experimental groups even though Mn was added to commercial pellets (as documented in Supplementary Material Figure S2). Given that bone is a pool for Mn with greater than 40% of the total Mn content of the body, its deficiency can lead to disorders of growth and bone development in animals and osteoporosis in humans. It could interfere with normal bone development (bone matrix synthesis and calcification) by inhibiting osteoblast viability [67]. In a study on rats, a deficient diet (low or depleted Mn and/or Cu) resulted in lower serum and femur Mn and/or Cu levels but concomitantly increased serum and decreased bone Ca levels [68], which was not the case in our study. Some fungi and bacteria are known to biodegrade BPA with ligninolytic enzymes, such as lignin peroxidase, Mn peroxidase (MnP), and laccase, including *Bacillus* sp. from the human gut microbiota [69,70], or catalases, including manganese catalase [71]. In addition, MnP and laccase can degrade BPA and abolish its estrogenic effect [61]. However, to our knowledge, no studies have reported enzymatic activities with Mn as a cofactor associated with BPA in the intestinal or rumen microbiota [61]. We can postulate that the rumen microbiota in the present study (fungi and/or bacteria) could adapt to BPA in the feed and degrade it by manganese catalase or peroxidase. If the microbiota used Mn as a cofactor in the enzymatic reactions of BPA degradation from the feed or bone reserves, this could have resulted in a lower amount of Mn in the bones. Another speculation related to our results was that the rumen microbiota might degrade BPA to some extent, resulting in a lower amount of BPA that could potentially be reabsorbed into the body, leading to minimal or no (adverse) effects in the animals. Nevertheless, after dietary exposure, buccal absorption that bypasses the hepatic first-pass effect of BPA should be considered in ruminants [49]. Reports have shown that BPA undergoes rapid metabolism in the body (glucuronidation and sulfation in the liver), and only a small amount is excreted unchanged via the biliary route or urine. Moreover, greater than 90% of BPA is eliminated 24 h after ingestion [72]. To the best of our knowledge, BPA levels in bone tissue after dietary exposure in laboratory rodents or sheep are not known and remain speculative at this moment. Therefore, it is difficult to assess whether the concentrations in cellular studies or dosage in animal studies are realistic [72]. Given that no parameters or the ratio of buccal/digestive absorption were measured in this study to confirm either of our hypotheses, these questions remain open and pointed to a complex microbial mechanism and complexity of ruminant digestive tract physiology that warrants further study.

Our data suggest that the rumen microbiota and Mn may play a role in BPA metabolism. However, Mn depletion in bones did not affect bone metabolism or bone behavior during our study. Therefore, an extension of the study into adulthood might reveal its potential long-term effects on bone histometric and mechanical properties. Future studies should also consider including bone resorption markers from urine in addition to bone markers from serum. Histological techniques have rarely been used to assess the effects of BPA on bone tissue and EGP, although they may provide additional insight to better understand BPA-induced responses in bone tissue.

## 5. Conclusions

Our data showed that a 2-month dietary exposure of 25 µg BPA/kg bw/day did not appear to affect the geometry, histomorphology, metabolism, and biomechanical behavior of femurs of growing rams, except for the bone composition of microelements (Mn, Cu). One of the possible explanations for the changes in Mn and Cu in bone could include the potential involvement of rumen microbiota in BPA metabolism. Future studies are, therefore, needed to elucidate the mechanism of BPA metabolism in the rumen and its impact on bone health in polygastric animals, as sheep represent a possible alternative model for orthopedic studies in humans.

## Figures and Tables

**Table 1:** Serum concentration of bone turnover markers of rams in the BPA and control groups. The BPA group received 25 µg BPA/kg bw/day for 64 days. Values are given as least square means, except in CTX-I and P1NP, where the relative values were used.

BPA Application (*n* = 7/Group) ^#^	Before		Day 31		Day 64		SEM	P-Time	P-Group	P-Interaction
	Control	BPA	Control	BPA	Control	BPA				
Urea (mmol/L)	5.31 ^ab^	5.36 ^ab^	5.87 ^b^	5.93 ^b^	4.18 ^a^	5.31 ^ab^	0.38	0.017	0.196	0.274
Creatinine (µmol/L)	59.7	59.7	52.3	51.0	53.7	49.1	2.95	0.011	0.423	0.729
Cholesterol (mmol/L)	1.81	2.01	1.71	1.70	1.71	1.84	0.10	0.150	0.231	0.589
AST (µkat/L)	2.21 ^ab^	2.61 ^b^	1.82 ^a^	1.96 ^a^	1.81 ^a^	2.07 ^ab^	0.14	<0.001	0.022	0.659
GGT (µkat/L)	0.90 ^a^	1.03 ^ab^	0.98 ^a^	1.10 ^ab^	1.04 ^ab^	1.23 ^b^	0.05	0.008	0.002	0.792
Ca (mmol/L)	2.59	2.49	2.60	2.51	2.65	2.63	0.05	0.095	0.120	0.697
PO_4_ (mmol/L)	2.79 ^b^	3.11 ^b^	2.65 ^ab^	2.88 ^b^	2.18 ^a^	2.64 ^ab^	0.12	<0.001	0.001	0.645
Ca:PO_4_	0.93 ^b^	0.82 ^b^	0.99 ^b^	0.89 ^b^	1.23 ^a^	1.01 ^ab^	0.05	<0.001	0.002	0.460
CTX-I ^#^ (pg/mL)	1 ^a^	1 ^a^	1.58 ^ab^	1.21 ^ab^	2.21 ^b^	1.66 ^ab^	0.28	0.008	0.184	0.603
P1NP (ng/mL)	1	1	2.40	1.03	2.10	1.01	0.67	0.537	0.143	0.563

^#^ Except parameter CTX-I (*n* = 6 animals/group). The statistical significance of differences between the groups was analyzed using the Proc Mixed function, including repeated measurements within the animal. BPA—bisphenol A, AST—aspartate aminotransferase, GGT—gamma-glutamyl transferase, CTX-I—C-terminal telopeptide of type I collagen, P1NP—N-terminal propeptide of type I procollagen. ^a,b^ Different superscript letters in each row indicate significant differences (*p* ≤ 0.05).

**Table 2:** Serum concentration of bone turnover markers of rams before the first BPA application compared with reference values for sheep from the manufacturer (MyBioSource, San Diego, CA, USA).

Bone Turnover Marker	Commercial Sheep Kit References			Our Study		
	Concentration Values	Age of Animals	Animal Sex	Before the First Application	Age of Animals	Animal Sex
CTX-I (pg/mL)	537–759	n.d.	n.d.	86–634 (*n* = 12)	9–10 months	12 males *
P1NP (ng/mL)	15.67–65.23	7–8 months	15 females, 15 males	28–158 (*n* = 14)	9–10 months	14 males

* Two samples had to be excluded due to hemolysis. CTX-I—C-terminal telopeptide of type I collagen, P1NP—N-terminal propeptide of type I procollagen; n.d.—data not provided by the manufacturer.

**Table 3:** Femur geometry (left femurs) and histomorphometry of EGP (right femurs) of rams in the BPA and control groups at the end of the study. The BPA group received 25 µg BPA/kg bw/day for 64 days. Values are given as the mean ± SD.

Femur Geometry	Control (*n* = 7)	BPA (*n* = 7)	*p*-Value
Relative femur mass (%)	0.372 ± 0.032	0.390 ± 0.038	0.356
Femur length (mm)	198 ± 7	198 ± 6	0.906
Femur width (mm)	20.8 ± 1.1	20.3 ± 0.8	0.355
Total bone area (cm^2^)	3.12 ± 0.29	3.04 ± 0.19	0.546
Cortical bone area (cm^2^)	1.53 ± 0.26	1.56 ± 0.17	0.831
ctCSA/tCSA	0.49 ± 0.04	0.51 ± 0.05	0.368
Cortical bone density (HU)	413 ± 72	423 ± 57	0.774
Trabecular bone density (HU)	1821 ± 47	1804 ± 43	0.515
BV/TV	0.78 ± 0.07	0.79 ± 0.09	0.690
Tb.Th (mm)	4.30 ± 1.58	5.31 ± 2.44	0.378
Tb.Sp (mm)	1.25 ± 0.20	1.29 ± 0.13	0.596
DA	0.31 ± 0.06	0.37 ± 0.11	0.264
EGP histomorphometry	Control (*n* = 6)	BPA (*n* = 7)	*p* value
EGP thickness (µm)	398 ± 31	405 ± 40	0.735
RZ thickness (%)	14.14 ± 2.63	14.17 ± 3.01	0.982
PZ thickness (%)	43.26 ± 5.63	42.24 ± 3.96	0.709
HZ thickness (%)	35.53 ± 4.58	35.83 ± 4.61	0.910
CZ thickness (%)	7.07 ± 1.01	7.76 ± 1.64	0.392

The statistical significance of differences between the groups was analyzed by independent samples *t*-test. *p* ≤ 0.05 vs. the control group. EGP—epiphyseal growth plate, BPA—bisphenol A, CSA—cross-sectional area, ct—cortical, tCSA—total CSA, HU—Hounsfield units, BV/TV—bone volume fraction, Tb.Th—trabecular thickness, Tb.Sp—trabecular separation, DA—degree of anisotropy, RZ—zone of reserve cartilage, PZ—zone of proliferation, HZ—zone of hypertrophy, CZ—zone of calcified cartilage.

**Table 4:** Biomechanical parameters and mineral content of the left femur of rams in the BPA and control groups at the end of the study. The BPA group received 25 µg BPA/kg bw/day for 64 days. Values are given as the mean ± SD.

Parameter/Group	Control (*n* = 7)	BPA (*n* = 7)	*p*-Value
Max force (kN)	3.11 ± 0.44	3.22 ± 0.71	0.734
Displacement (mm)	2.52 ± 0.34	2.47 ± 0.25	0.777
Stiffness (N/mm)	1259 ± 269	1291 ± 200	0.803
Work to fracture (Nmm)	3912 ± 760	4028 ± 1257	0.838
Bending strength (MPa)	163 ± 21	169 ± 25	0.654
Modulus of elasticity (GPa)	7.87 ± 0.59	8.36 ± 1.19	0.351
Ash (g/kg of dry bone)	201.1 ± 22.2	194.5 ± 21.0	0.570
Ca (g/kg ash)	361.7 ± 6.66	350.2 ± 14.07	0.073
P (g/kg ash)	168.0 ± 1.11	164.4 ± 6.52	0.192
Ca:P ratio	2.15 ± 0.05	2.13 ± 0.01	0.292
Mg (g/kg ash)	7.64 ± 0.397	7.39 ± 0.545	0.359
Zn (mg/kg ash)	184.7 ± 23.9	187.1 ± 20.1	0.843
Fe (mg/kg ash)	137.1 ± 27.2	118.1 ± 24.1	0.192
Mn (mg/kg ash)	26.73 ± 8.47 ^a^	13.43 ± 0.959 ^b^	0.006
Cu (mg/kg ash)	11.93 ± 1.43 ^a^	14.96 ± 2.12 ^b^	0.011

The statistical significance of differences between the groups was analyzed by independent samples *t*-test. *p* ≤ 0.05 vs. the control group. BPA—bisphenol A. ^a,b^ Different superscript letters in each row indicate significant differences (*p* ≤ 0.05).

## Data Availability

The data presented in this study are available on request from the corresponding author.

## Supplementary Materials

The following supporting information can be downloaded at https://www.mdpi.com/article/10.3390/ani12172179/s1, Supplementary Table S1: Values of vital signs and serum biochemistry; Supplementary Material Figure S2: A certificate of ingredients and chemical composition of commercial pellets Schafkorn.
