# Supplementary material for: Experimental Exposure to Bisphenol A Has Minimal Effects on Bone Tissue in Growing Rams—A Preliminary Study
Source: Animals (Basel). 2022 Aug 25;12(17):2179. doi: 10.3390/ani12172179 (PMC9454980; doi:10.3390/ani12172179)
Supplement: Supplementary file 1 [file animals-12-02179-s001.zip › supplementary material_S2_feed_certificate.pdf]

Material S2: A certificate of ingredients and chemical composition of commercial pellets Schafkorn, Raiffeisengenossenschaft Osttirol (Lienz, Austria).

GARANT QUALITÄTSFUTTER

UNSER LAGERHAUS
WARENHANDELS Ges.m.b.H.

A-9020 Klagenfurt
Südring 240
Postfach 605
Tel.: +43 (0) 463 / 3865 - 0
Fax: +43 (0) 463 / 3865 - 578

UNSER LAGERHAUS

Bediener: **Systemverwalter**
Datum / Zeit: **20.03.2017 / 12:09:51**
Station: **KESASRV01**

VETERINARSKA FAKULTETA
CSR VREMSCICA

GERBICEVA 60
1000 LJUBLJANA

Telefon:
Fax:
UID-Nummer: -
Auftrag: -
Bestellung: 620318.1
Bestell-Datum: 20.03.2017
Liefertermin: 20.03.2017
Wiegedatum: 20.03.2017 12:09
Kundennummer: 472964
LFBIS-Nr:
Kraftfahrzeug: H1

Rückstellmusternummer:

LIEFERSCHEIN / No. LOTTO: 620318.1
WS-NUMMER: 37

ARTIKEL-NR	ARTIKELBEZEICHNUNG	MENGE	KESSEL	WIEGE-NR	WIEGE-DATUM
50767	Schafkorn Lac	8540 kg	3,4	37	20.03.2017

Info: PASTUS+ AMA Gütesiegel tauglich

Erstverwiegung: Brutto 18260 kg, Tara 0 kg, 20.03.2017 11:55
Zweitverwiegung: Brutto 26800 kg, Tara 18260 kg, 20.03.2017 12:09

Schaf Korn Lac

Ergänzungsfuttermittel für Schafe, Lämmer und Widder /Popolna krmna mešanica za ovce, vzrejo in pitanje jagnet in ovnov

Inhaltsstoffe /Sestavine

17,50 % Rohprotein /surove beljakovine
2,50 % Rohfett /surova olja in maščobe
6,40 % Rohfaser /surove vlaknine
9,60 % Rohasche /surov pepel
1,65 % Calcium /kalcij
0,65 % Phosphor /fosfor
0,50 % Natrium /natrij

Zusatzstoffe je kg /Dodatki snovi na kg

ernährungsphysiologische Zusatzstoffe / prehranski dodatki

13.500I.E. Vitamin A (3a672a) Retinylacetat / , 1.600I.E. Vitamin D_3 (E 671) / , 100mg Vitamin E (E 3a700 all-rac- α Tocopheryl - acetat) / , 10mg Eisen-(II)-sulfat, Monohydrat (E1) /železovega sulfata, Monohidrata (E1), 2mg Mangan-(II)-oxid (E5) /manganovega oksida (E5), 6mg Zinkoxid (3b603) /cinkovega oksida (3b603) , 0,5mg Natriumselenit (E8) /natrijevegaselenita (E8)

Zusammensetzung /Sestava

Gerste /ječmen, Rapsextraktionsschrotfutter /r kleie /rženi otrobi, Weizenkleie /pšenični otrobi, Trockenschlempe, (Mais, Weizen) /suhe koruz ter /koruzni gluten (19%), Melasse /melasa, Ca karbonat, Zuckerrübentrockenschnitzel /suhi p rid /natrijev klorid

Fütterungshinweise /Navodila za uporabo

Die Futtermenge richtet sich nach Größe und I nach Milchleistung und Qualität des wirtschafts Futters.Milchschafe:0,3 - 1,8 kg/Tier/Tag --Wid der Decksaison 0,2 - 0,5 kg/Tier/Tag außerhal Lämmermast:0,6 - 1,0 kg/Tier/Tag je nach Gru ovce: 0,3 - 1,8 kg/žival na dan, ovni: 0,5 - 1,2 k parjenja ostale živali: 0,2 - 0,5 kg/žival na dan, pitanje:0,6 - 1,0 kg/žival na dan.

Nettomasse: siehe Sackaufdruck bzw. lose od schein /neto teža: vreča, rinfuza ali big bag od

Mindestens haltbar bis 6 Monate nachdem He Herstellungsdatum: siehe Sackaufdruck od. Li uporaben najmanj 6 mesecev od datuma proi proizvodnje: odtisnjen na embalaži!

pastus®	AMA-Gütesiegel tauglich

Hersteller/Proizvajalec in oseba odgovorna za označevanje: UNSER LAGERHAUS Warenhandels Ges. M. b. H., Kla 240, 0463/3865-671 Austria, Zulassungs.Nr.: αAT 5003 Vertreiber/Distributer RAIFFEISEN d.o.o., Industrijska 8, 22 3865 671 /00386 272 00 556 .

Reinigungsbestätigung

Hiermit wird bestätigt, dass die Ladefläche bzw. die Silokammer des LKW's bzw. des LKW-Anhängers vor dem Transport von Futtermittel laut Lieferschein

GARANT GARANTIERT DAS BESTE FUER IHR TIER /GARANT - ZAGOTAVLJA NAJBOLJŠE Z

vom Beladeort: siehe Lieferschein nach Entladeort: siehe Lieferschein rückstandslos gereinigt wurde.

Der für den Transport verantwortliche Fahrer/Spediteur bestätigt die Richtigkeit der gemachten Angaben und nimmt zur Kenntnis, dass diese Bestätigung am Verladeort aufzubewahren ist. Der Verlader bestätigt, dass die vom Fahrer/Spediteur gemachten Angaben mittels optischer Kontrolle nach bestem Gewissen überprüft wurden.

Unterschrift Fahrer .... Datum .... Verlader ....

Die Ware bleibt bis zur vollständigen Bezahlung unser Eigentum.

Der Käufer nimmt die Allgemeinen Geschäftsbedingungen der Unser Lagerhaus Warenhandelsges.m.b.H., die in den Geschäftsräumlichkeiten aufliegen und unter www.unser-lagerhaus.at veröffentlicht sind, zustimmend zur Kenntnis, womit diese Vertragsinhalt werden. Bei Zahlungsverzug werden 12,00 % bankmäßige Verzugszinsen ab Fälligkeitstag mit vierteljährlicher Belastung verrechnet. Ausschließlich zuständig für alle sich mittelbar oder unmittelbar aus einem Geschäft ergebenden Streitigkeiten ist das für den Sitz unserer Gesellschaft sachlich zuständige Gericht (gilt nicht für Verbrauchergeschäfte). Für Verbrauchergeschäfte wird die Zuständigkeit österreichischer Gerichte vereinbart.

Unser Lagerhaus Warenhandelsges.m.b.H., Südring 240, 9020 Klagenfurt, Niederlassung Tirol: Duilestraße 20, 6021 Innsbruck Sitz der Gesellschaft: 9020 Klagenfurt, Gerichtsstand Klagenfurt, FB-Gericht: LG Klagenfurt, FB-Nummer: 109975d, UID: ATU25323307, DVR: 0662500, AT-BIO-902, NICHT VEREINBARTE SKONTOABZÜGE WERDEN NICHT ANERKANNT! Bankverbindung: Raiffeisen Landesbank Kärnten, BLZ 39000, Kto. 1.093.566, IBAN AT17 3900 0000 0109 3566, BIC RZKTAT2K
