# Supplementary material for: Experimental Exposure to Bisphenol A Has Minimal Effects on Bone Tissue in Growing Rams—A Preliminary Study
Source: Animals (Basel). 2022 Aug 25;12(17):2179. doi: 10.3390/ani12172179 (PMC9454980; doi:10.3390/ani12172179)
Supplement: Supplementary file 1 [file animals-12-02179-s001.zip › Supplementary Table S1_vital_signs.pdf]

Table S1: Values of vital signs and serum biochemistry of rams before the animals were administered BPA. Values are given as the mean ± SD.

**Parameter *(n=14)***	**Value**
**Body temperature (°C)**	39.8 ± 0.3
**Pulse (beats/min)**	68 ± 1
**Respiratory rate (breaths/min)**	36 ± 5
**Ruminations in 2 min**	2
**Urea (mmol/L)**	5.3 ± 0.7
**Ca (mmol/L)**	2.5 ± 0.1
**PO_4 (mmol/L)**	2.95 ± 0.4
**Creatinine (µmol/L)**	59.7 ± 9.5
**Cholesterol (mmol/L)**	1.91 ± 0.30
**Triglycerides (µmol/L)**	357 ± 102
**AST (µkat/L)**	2.41 ± 0.41
**GGT (µkat/L)**	0.962 ± 0.142

Ca – calcium, PO_4 – inorganic phosphate, AST – aspartate aminotransferase, GGT – gamma glutamyl transferase.
